# Sodium Hypochlorite (NaClO) Disturbed Lipid Metabolism in Larval Zebrafish (*Danio rerio*), as Revealed by Lipidomics and Transcriptomics Analyses

**DOI:** 10.3390/toxics12100718

**Published:** 2024-09-30

**Authors:** Wen Wang, Hua Yang, Xingning Xiao, Qu Chen, Wentao Lv, Lingyan Ma, Chanlin Fang, Yuanxiang Jin, Yingping Xiao

**Affiliations:** 1 State Key Laboratory for Managing Biotic and Chemical Threats to the Quality and Safety of Agro-Products, MOA Laboratory of Quality & Safety Risk Assessment for Agro-Products (Hangzhou), Institute of Agro-Product Safety and Nutrition, Zhejiang Academy of Agricultural Sciences, Hangzhou 310021, Chinaxingningxiao@126.com (X.X.); cq18936549428@163.com (Q.C.); lvwt@zaas.ac.cn (W.L.);; 2 College of Biotechnology and Bioengineering, Zhejiang University of Technology, Hangzhou 310032, China

**Keywords:** NaClO, lipid metabolism, lipidomic analysis, transcriptomic analysis, zebrafish

## Abstract

Sodium hypochlorite (NaClO) has been widely utilized since the initial outbreak of coronavirus disease (COVID-19). The widespread use of NaClO means that it can directly enter aquatic ecosystems through wastewater discharge. In this study, we analyzed the expression of *PPAR-γ*, *FAS*, and *ACC1*, which significantly increased in larval zebrafish following exposure to 300 μg/L NaClO for 7 days. Additionally, we examined the effects of high concentrations of NaClO on zebrafish through non-targeted lipidomics and transcriptomics. A total of 44 characteristic lipid molecules were identified using non-targeted lipidomics; an absolute quantitative analysis revealed that the contents of these subclasses of lipids decreased significantly following exposure to 300 μg/L NaClO for 7 days. The levels of triglyceride (TG), phosphatidylethanolamines (PE), and diglyceride (DG) were particularly affected. Transcriptomic analysis revealed that exposure to 300 μg/L NaClO could significantly disrupt global gene transcription in larval zebrafish. Interestingly, more than 700 differentially expressed genes (DEGs) were identified, primarily associated with lipid metabolism and glycometabolism pathways. Overall, our study provided new insights into the toxicological effects of chlorine-containing disinfectants in aquatic organisms.

## 1. Introduction

In 2019, coronavirus disease (COVID-19) broke out and was later declared a global pandemic on 11 March 2020. The severe acute respiratory syndrome coronavirus-2 (SARS-CoV-2) disease is transmitted through the inhalation of virus particles suspended in the air or through indirect contact with contaminated objects [1,2]. To reduce the spread of infection, public places, including hospitals, must be thoroughly disinfected. Disinfection is a critical process for eliminating pathogenic microorganisms and reducing disease transmission [3]. Chlorine-based disinfectants are recommended as oxidative disinfectants for preventing the spread of SARS-CoV-2 [4]. As a precaution, many institutions have increased the use of chlorine in wastewater disinfection to achieve residual free chlorine concentrations above 6.5 mg/L [5]. Evidence indicates that SARS-CoV-2 can be inactivated at residual chlorine concentrations exceeding 0.5 mg/L [6]. Reports have shown that the concentration of residual chlorine in treated wastewater from treatment plants ranged between 0.09 and 8.5 mg/L [7,8]. Data indicated that China has distributed 2000–5000 tons of disinfectants in Wuhan since the outbreak of the epidemic in 2020 [6]. These chlorine-based compounds can directly enter both aquatic and terrestrial environments, including via wastewater discharge [5]. Direct runoff and indirect sewage discharge significantly harm the water system [9]. Previous studies have shown that chlorine disinfectants primarily damage plants by destroying cell walls or proteins, and the generation of by-products can lead to the death of aquatic organisms [6,9]. However, the specific effects of disinfectants on aquatic organisms remain largely unknown.

Lipids, fundamental components of various organelles, are crucial for numerous metabolic and physiological processes [10]. Multiple studies have suggested that environmental pollution disrupts lipid metabolism, resulting in the accumulation of fat in the liver [11,12,13,14]. Excessive fat accumulation can damage fish by inhibiting growth and reducing energy utilization, ultimately diminishing their tolerance to external environmental stresses [15]. Lipid metabolism in fish closely resembles fat metabolism in mammals [16,17,18]. Some studies have indicated that triazole fungicides can cause lipid metabolism disorders, but not whether disinfectants produce similar effects [8,19,20]. Furthermore, Braun et al. (2017) [21] and Heindel et al. (2017) [22] have reported that exposure to environmental pollutants can induce obesity and metabolic disturbances in mammals. Zhang et al. (2020) [6] recently reported that residual chlorine disrupts microbial communities and promotes the spread of antibiotic resistance in zebrafish. However, due to the limited impact of chlorine-containing disinfectants on lipid metabolism in zebrafish, it is necessary to clarify the relationship between the liver injury induced by these disinfectants and lipid metabolism, as well as other related toxicological effects.

The zebrafish is a widely used model organism in genetics, developmental biology, and ecotoxicology research [23,24]. It is highly valued due to its 87% genomic homology with humans and the similarity of its developmental molecular mechanisms to those of mammals [25,26]. Additionally, zebrafish embryos are transparent, offering high optical clarity [27,28], which allows for the detailed observation of organ development using microscopy. Given these characteristics, zebrafish were chosen as the experimental subject of this study. Therefore, we selected larval zebrafish as the model organism, and the animals were exposed to the compound for 7 days. Non-targeted lipidomics was employed to analyze lipid content changes. Transcriptomic analysis was conducted to identify differentially expressed genes (DEGs), followed by Kyoto Encyclopedia of Genes and Genomes (KEGG) pathway enrichment analysis. Additionally, the expression of lipid metabolism-related genes was examined using real-time quantitative PCR (RT-qPCR) to further elucidate the associated biological processes and underlying mechanisms. These findings provide insights into the toxic effects of NaClO on aquatic organisms and suggest the need for appropriate measures to regulate water discharge and disinfectant use based on these results.

## 2. Materials and Methods

### 2.1. Reagents and Chemicals

NaClO (7681-52-9) was obtained from the Shanghai Bioengineering Institute (Shanghai, China), with an initial chlorine concentration of 56.8 mg/mL. For each experiment, we prepared a 200 mg/L NaClO solution for exposure and working solutions diluted in ultrapure (UP) water (200 μL stock solution + 50 mL UP water).

### 2.2. Zebrafish Maintenance and Spawning

Wild-type (WT) zebrafish were reared in aerated water, with the relevant indices referenced from our previous study [29]. Zebrafish were maintained under a photoperiod of 14 h light/10 h dark at 28 ± 1 °C and fed on freshly hatched brine shrimp twice a day. Male and female zebrafish were placed in glass jars at a ratio of 2:1 at 20:00. The next morning, at 8:00, we turned on the light and turned off the pumps, respectively, and fertilized eggs were collected for exposure. The embryos were collected following the standard protocols described by Westerfield (1993) [30].

### 2.3. Embryonic Exposure

A total of 120 healthy embryos were randomly selected and placed into a 500 mL glass beaker with 200 mL of exposure solution. The exposure solutions were prepared at concentrations of 30, 100, and 300 μg/L, representing low, medium, and high concentrations, respectively. During the exposure period, the solution was refreshed at 8:00 and 20:00. The exposure time of zebrafish juveniles was 7 days and without any food given to larval zebrafish. Each group comprised six replicates, and samples were stored at −80 °C for physiological assessments. For lipidomics analysis, 300 larval zebrafish were collected for testing, with five parallels established. Following our previous experience, a total of 120 zebrafish larvae were collected for total RNA extraction, which was utilized for transcription analysis.

### 2.4. Physiological Index Analysis

According to the ratio of fish:PBS = 1:1, each sample was ground using a grinder (Jinxing, Shanghai, China). The mixture was then centrifuged at 4000 rpm for 10 min and the supernatant was collected for subsequent experiments. The content of total cholesterol (TCHO), triglyceride (TG), glucose (GLU), pyruvic acid (PYR), and low-density lipoprotein (LDL) were measured according to the instructions provided by the Nanjing Jiancheng Bioengineering Institute. The concentration of protein was qualified using abicinchoninic acid (BCA) assay kits (Beyotime, Shanghai, China).

### 2.5. Gene Expression Analyses

The total RNA from 20 larval zebrafish in each replication was extracted with a TRIzol reagent (Vazyme, Nanjing, China). The quality and purity of the RNA were assessed, based on A260/280 (1.8–2.0) and A260/230 (≥2.0) ratios. The qualified RNA was then reverse-transcribed to cDNA using reverse transcription kits (Vazyme, Nanjing, China). Subsequently, real-time quantitative PCR (RT-qRCR) analysis was performed using the SYBR Green system (Vazyme, Nanjing, China). The primer sequences of the genes used in this study are listed in Table S1. RT-qPCR was performed as previously described [31], under the following reaction conditions: 3 min at 95 °C, followed by 39 cycles of 15 s each at 95 °C and 60 s at 60 °C. The housekeeping gene was EF1α, and the quantification of the relative abundance of gene transcript was performed using the 2^−ΔΔCt^ method. It is important for PCR efficiency to be close to 100% (E = 2) when employing the delta-delta Ct method for data analysis [32]. In our experiment, the amplification coefficient was close to 2.

### 2.6. Nontargeted Absolute Quantitative Lipidomic Analysis

Lipidomics is a method for systematically analyzing changes in lipid composition and their expression in living organisms using high-throughput analytical techniques. In this study, various lipids were analyzed by non-targeted analysis and absolute quantification. We employed a non-targeted lipidomics platform based on the UPLC-Orbitrap mass spectrometry system, which was combined with LipidSearch (Thermo Scientific^TM^, Waltham, MA, USA) and 14 types of paper molecular isotope internal standards for lipid identification and data preprocessing. The absolute contents of paper molecules in the samples were obtained on a large scale [33]. The entire experiment consisted of sample preprocessing, mass spectrometry analysis, and data analysis. Details regarding sample pretreatment time and mass spectrometry conditions are provided in the Supplementary Materials.

The quality of the data extracted using LipidSearch was evaluated and analyzed. Data analysis primarily involved identification, quantitative statistics, lipid composition analysis, and the differential expression analysis of lipids. The corresponding lipid concentrations were assessed through absolute quantitative lipidomics.

### 2.7. Transcriptomic Analysis

The total RNA was extracted using TRIzol reagent, and its integrity was assessed with the RNA Nano 6000 Assay Kit of the Bioanalyzer 2100 system (Agilent Technologies, Santa Clara, CA, USA). The NEBNext^®^ UltraTM RNA Library Prep Kit for Illumina^®^ (New England Biolabs, Ipswich, MA, USA) was employed to construct libraries for sequencing. The Qubit2.0 Fluorometer and qRT-PCR were utilized to accurately quantify the libraries. Following the manufacturer’s instructions, clustering of the index-coded samples was performed on the cBot Cluster Generation System using the TruSeq PE Cluster Kit v3-cBot-HS. Finally, after cluster generation, the library preparations were sequenced on an Illumina Novaseq platform, generating 150 bp paired-end reads.

The sequenced fragments were converted into FASTQ format, using CASAVA for base recognition. To ensure the quality and reliability of the analysis, the raw data were filtered, and adaptors, as well as low-quality reads and ambiguous bases (N), were removed. Differential expression analysis was conducted using DESeq2 software (version 1.20.0). A corrected *p*-value of <0.05 and a fold change of ≥2 were established as criteria for significant differentially expressed genes (DEGs). Finally, gene ontology (GO) and KEGG enrichment analyses for DEGs were performed using the clusterProfiler package in R [14].

### 2.8. Statistical Analysis

All experiments were conducted in six replicates, and the results are represented as mean ± SEM. A one-way ANOVA was performed, with *p* < 0.05 (*) and *p* < 0.01 (**) indicating statistically significant differences. For multi-omics analysis, the data were screened and plotted based on *p*-values and fold changes.

## 3. Results

### 3.1. Effects of NaClO Exposure on the Physical Index

As shown in Table 1, the level of GLU decreased significantly (*p* < 0.05) after being exposed to 100 and 300 μg/L NaClO, and the PYR level also decreased significantly after exposure to 300 μg/L NaClO. In contrast, the levels of TG, TC, and LDL showed no significant changes.

### 3.2. Effects of NaClO Exposure on Gene Transcription Related to Energy Metabolism

Based on the detection of physical indices, we further evaluated the expression of genes related to energy metabolism using RT-qPCR analysis to provide deeper insights into metabolic processes.

**Figure 1:**
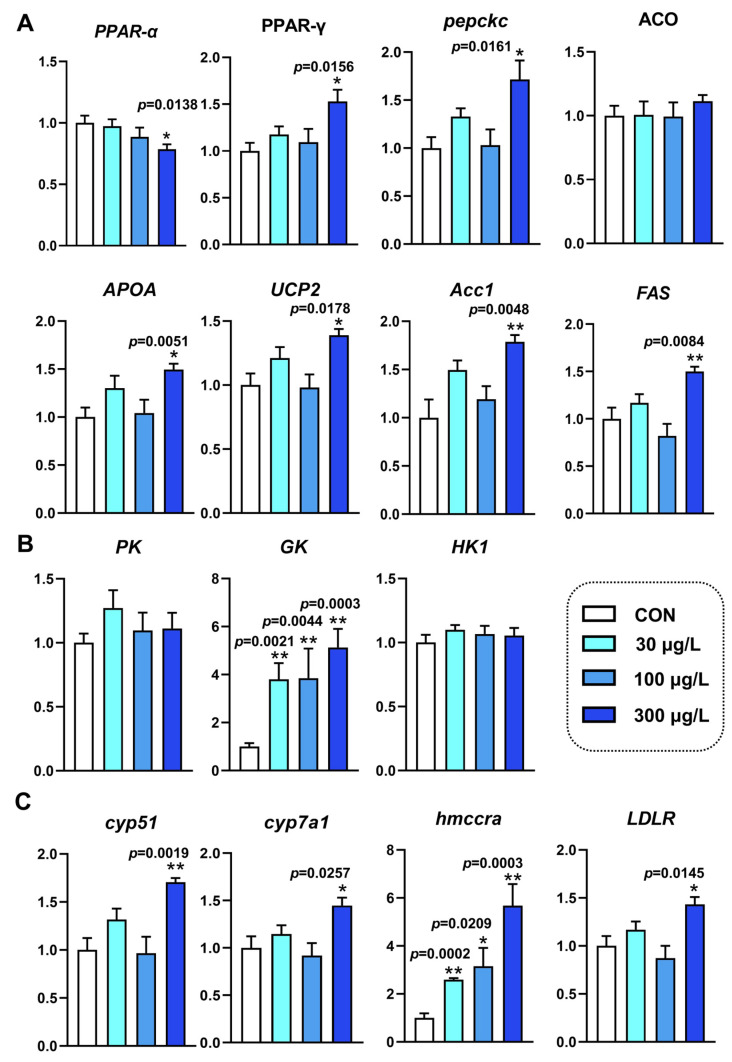
Effects of NaClO exposure on the mRNA expression of glucolipid metabolism-related genes in zebrafish larvae. (**A**) Expression of lipid-related genes. (**B**) Expression of glycolysis-related genes. (**C**) Expression of cholesterol-related genes. Differences between treated and control groups are analyzed by a one-way ANOVA and presented as the mean ± SEM (n = 6; * *p* < 0.05; ** *p* < 0.01).

As shown in Figure 1A, exposure to NaClO significantly affected the expression levels of the genes involved in lipid metabolism. The transcription of peroxisome proliferator-activated receptor-γ (*PPAR-γ*), cytosolic phosphoenolpyruvate carboxy kinas (*PEPckc*), apolipoprotein A-IV (*APOA*), uncoupling protein 2 (*UCP2*), acetyl-CoA carboxylase 1 (*Acc1*), and fatty acids (*FAS*) increased significantly following exposure to high concentrations of NaClO (*p* < 0.05). Conversely, the mRNA levels of peroxisome proliferator-activated receptor-α (*PPAR-α*) significantly decreased in the group treated with 300 μg/L NaClO (*p* < 0.05).

The transcription of genes related to glucose metabolism was also altered in zebrafish larvae following NaClO exposure. Notably, the relative abundance of glucokinase (*GK*) in the treated group was significantly higher than that in the control group (Figure 1B) (*p* < 0.05), while NaClO exposure had no significant effect on the transcription of pyruvate kinase (*PK*) and hexokinase (*HK1*).

Cholesterol is a key component of lipids. The mRNA expression levels of cytochrome P450 family 51 (*CYP51*), cholesterol 7α-hydroxylase (*CYP7A1*), hydroxymethyl glutaryl coenzyme A reductase a (*HMGCRa*), and low-density lipoprotein receptor a (*LDLR*) were assessed, revealing a significant upward trend in the high-concentration treatment group (*p* < 0.05).

### 3.3. Lipidomic Analysis of Larval Zebrafish Exposed to NaClO

**Figure 2:**
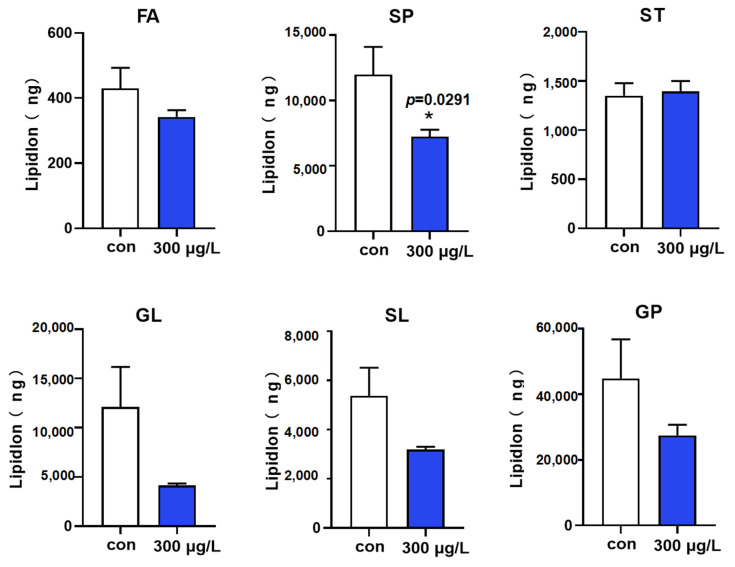
Changes in the content of the six lipid classes after NaClO exposure. (FA represents fatty acyls; GL represents glyceride, GP represents glycerophospholipids, SP represents sphingolipids, ST represents sterol lipids, and SL represents saccharolipids) (* *p* < 0.05).

The 44 identified characteristic metabolites were categorized into six classes of lipids: fatty acyls (FA), glyceride (GL), glycerophospholipids (GP), sphingolipids (SP), sterol lipids (ST), and saccharolipids (SL). As shown in Figure 2, the absolute contents of these lipid classes were statistically analyzed. The levels of various lipids (FA, SP, GL, SL, and GP) decreased after exposure to NaClO; however, some of these changes were not statistically significant. Correlational analysis was performed to assess the degree of metabolic similarity between significantly different lipids, enhancing our understanding of their mutual regulatory relationships during changes in biological states. The correlational analysis of the 44 lipid molecules (Figures S1 and S2) revealed positive correlations, indicating their involvement in the same metabolic pathway. For instance, TG positively correlated with most lipids, suggesting that they originate from the same synthesis pathway.

**Figure 3:**
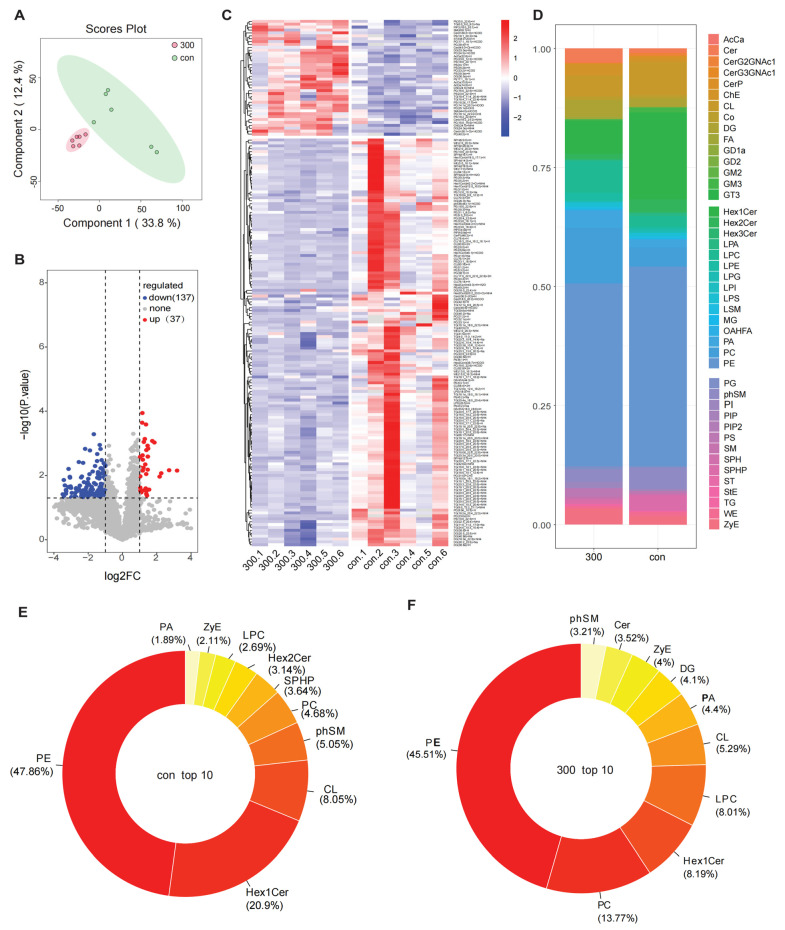
Lipidomic analysis and biological interpretation of the identified lipid metabolism molecular structure. (**A**) Two-dimensional PLS-DA score plot for lipid metabolites in larval zebrafish after treatment with NaClO. (**B**) Volcano plot of the biomarkers of lipid metabolism; (**C**) hierarchical cluster diagram of differentially expressed lipid molecules; (**D**) stacked bar graph of differentially expressed lipid molecules; (**E**) the top ten lipid molecules in the control group; (**F**) the top ten lipid molecules in the treatment group.

The untargeted lipidomic profiles of 12 zebrafish larvae samples were obtained by ultra-high-performance liquid chromatography-mass spectrum in series (UHPLC-MS) in both positive and negative ESI modes. A total of 1963 lipid species were identified. Following multivariate statistical analysis and data processing, a partial least-squares discriminant analysis (PLS-DA) was conducted (Figure 3A). The differential expression lipids between the two groups were clearly represented in the volcano plot, identifying 37 and 137 up-regulated and down-regulated lipids (*p* < 0.05 and fold change ≥ 2) following exposure (Figure 3B). These regulated lipids are displayed on a heatmap (Figure 3C). The repeatability within the group was good. To further analyze the altered lipids, stacked bar graphs were generated to illustrate lipid changes after exposure (Figure 3D). As shown in Figure 3D, the content of phosphatidylcholines (PC) decreased significantly, while the level of Hex1Cer increased significantly; the content of phosphatidylethanolamine (PE) remained unchanged. Subsequently, the top 10 lipids in the control and treatment groups were represented using pie charts (Figure 3E). As indicated in the previous histogram, PE exhibited the highest abundance, and its content did not change significantly after exposure.

The changes in the contents of lipid subclasses can reflect alterations in lipid functions. As shown in Figure S3A, the variations in each lipid molecule are illustrated in the pie chart (*p* < 0.05 and fold change ≥ 2), with TG levels being predominantly affected, reflecting changes in 55 lipid molecules. Furthermore, the alterations in PE and DG were secondary to TG, with 19 and 18 lipid molecules, respectively, exhibiting changes (Figure S3B). To comprehensively evaluate the rationale behind the differentially expressed lipids and to visually represent these changes after exposure, the expression of significant differential lipids (*p* < 0.05, fold change ≥ 4) was plotted on a bar chart (Table S2 and Figure S3C). Notably, the levels of several TG, PE, and cardiolipins (CL) decreased significantly following treatment compared to the control.

### 3.4. Transcriptomic Analysis after Exposure to NaClO

Given that lipid metabolism in zebrafish larvae was significantly affected by NaClO, we conducted a transcriptomic analysis to further investigate the effects of NaClO by comparing the high-concentration-treated group with the control group.

**Figure 4:**
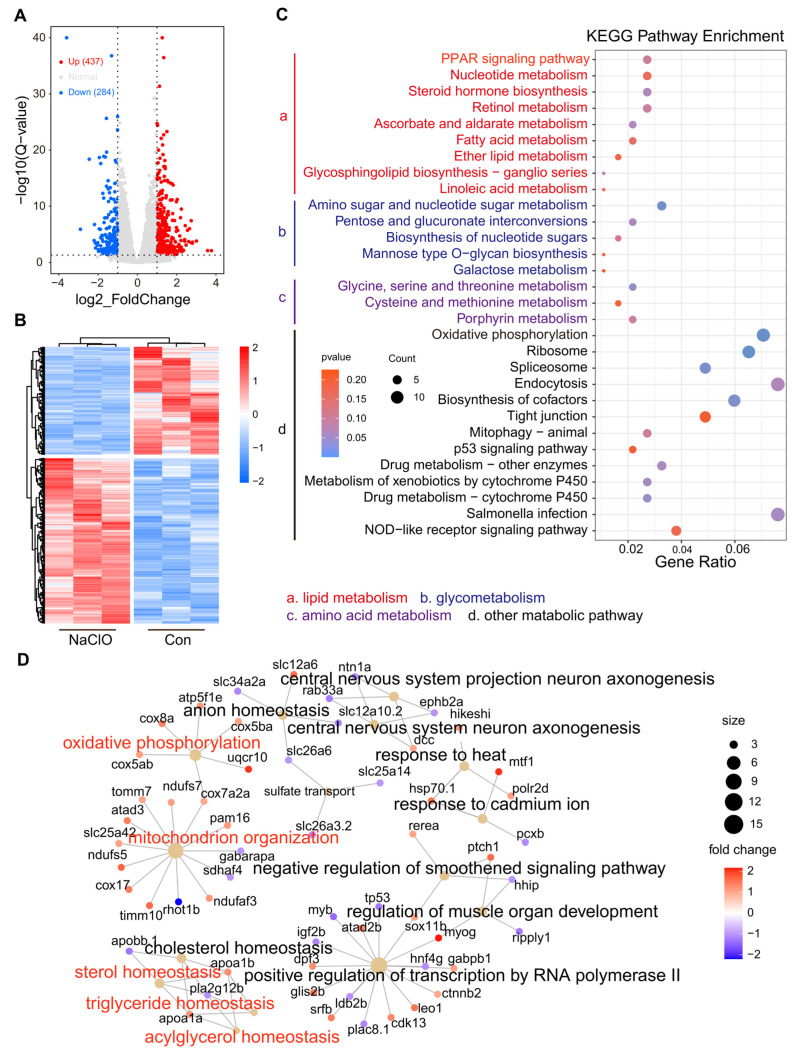
Transcriptomic analysis of gene expression in zebrafish larvae treated with NaClO for a week. (**A**) Volcano map of high concentration-treated group based on DEGs (Q-value ≤ 0.05 and fold change ≥ 1.5); (**B**) heatmap of cluster analysis using the FPKM of differentially expressed genes (DEGs) in the high-concentration-treated group; (**C**) scatter plots of KEGG functional enrichment, indicating the top 30 pathways based on DEGs in the high concentration-treated group; (**D**) the concept network of gene ontology (GO)-biological process (BP) terms related to DEGs in larval zebrafish after exposure to 300 μg/L NaClO for a week.

The DEGs between the two groups were clearly classified in the volcano plot, revealing 437 up-regulated and 284 down-regulated genes, respectively (*p* < 0.05 and fold change > 2) (Figure 4A). As illustrated in Fig. S4B, the heatmaps showed that all samples from the control and 300 μg/L NaClO-treated groups clustered together, indicating good repeatability (n = 3). To further investigate the effects of NaClO exposure on lipid metabolism in larval zebrafish, we conducted a KEGG functional enrichment analysis for the DEGs to elucidate the specific overrepresented metabolic pathways (Figure 4C). The most affected pathways were associated with energy metabolism, including those related to lipids, amino acids, and glucose. Additionally, the KEGG analysis indicated enrichment in other pathways related to drug metabolism and detoxification.

We conducted a GO enrichment analysis for DEGs, categorizing them into three main areas: biological process (BP), cellular component (CC), and molecule function (MF). The top 20 pathways were identified using a cut-off criterion of a *p*-value < 0.05 and are presented in bubble charts or histograms (Figure 4D and Figure S4). In the BP category, exposure to NaClO significantly impacted energy metabolism, including oxidative phosphorylation and sterol homeostasis (Figure 4D). In the CC and MF categories, the primary effects of NaClO exposure were observed in active transmembrane transporter activity, the mitochondrial membrane, and the mitochondrial inner membrane (Figure S4).

### 3.5. Enrichment of Genes Related to Lipid Metabolism and Transcriptome Verification

**Figure 5:**
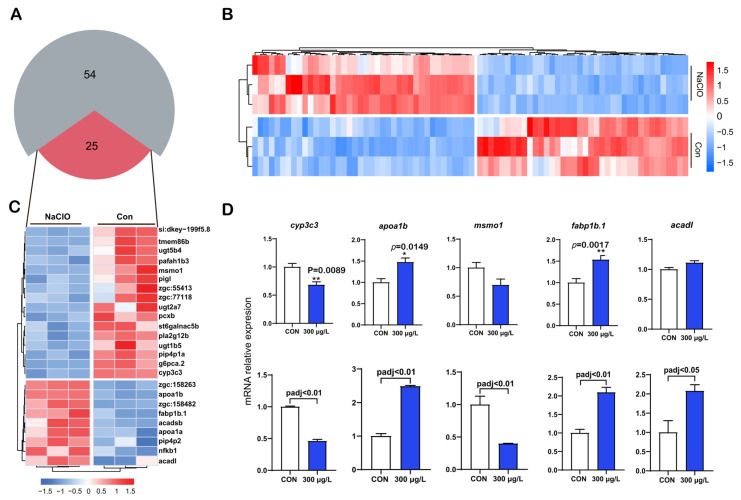
Genes related to lipid metabolism according to transcriptomics and the validation of these results. (**A**) The number of genes affected by NaClO exposure; (**B**) heatmap of the cluster analysis, including all genes related to lipid metabolism; (**C**) a total of 25 genes related to lipid metabolism were annotated; (**D**) several key genes associated with lipid metabolism were validated by qRT-PCR analysis. The first row represents the results of qRT-PCR; the second row represents the consistent results of the transcriptomic comparison (* *p* < 0.05; ** *p* < 0.01).

**Figure 6:**
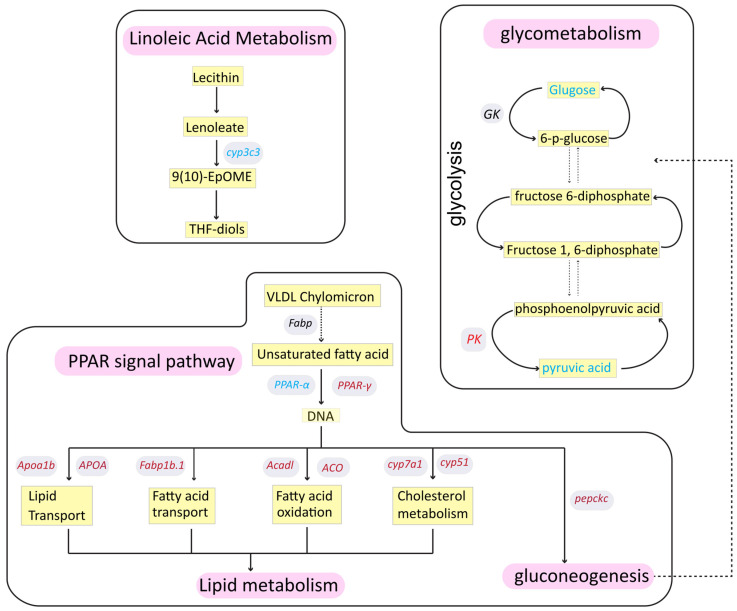
Summer figure of lipid metabolism. The yellow shading represents metabolites, and the gray shading represents genes. Red text represents a gene that is up-regulated, while blue text represents a down-regulated gene.

To further assess the genes involved in lipid metabolism that are affected by NaClO, we categorized the lipid metabolism-related genes and generated a pie chart (Figure 5A) and a heatmap (Figure 5B). As shown in Figure 5A, 79 genes related to lipid metabolism were identified, but only 25 could be annotated; among these, 9 were up-regulated and 16 were down-regulated. To validate these results, we randomly selected several key genes for further analysis. The results of the RT-qPCR analysis were consistent with those of transcriptomic analysis. The gene related to cytochrome P450-like (*cyp3c3*) decreased significantly after exposure (*p* < 0.05). Similarly, following NaClO exposure, the genes involved in cholesterol synthesis and transport, including methylsterol monooxygenase 1 (*msmo1*), exhibited a decreasing trend. Apolipoprotein A-Ib (*apoa1b*) and fatty acid binding protein 1b (*fabp1b.1*), which is related to lipid binding and transportation, were up-regulated after exposure to NaClO (*p* < 0.05). In contrast, the expression of acyl-CoA dehydrogenase long chain (*acadl*) remained unchanged, although the trend aligned with transcriptomic results. As illustrated in Figure 6, we summarized the RT-qPCR and transcriptomic analysis results, highlighting the relationships between several important genes and metabolites. Collectively, these findings indicate that NaClO exposure induced metabolic disorders, affecting lipid metabolism, linoleic acid metabolism, and glycometabolism.

## 4. Discussion

The COVID-19 pandemic necessitated the widespread use of disinfectants for wastewater treatment and site disinfection. The available data suggest that disinfection at pH < 8 and a free chlorine concentration of > 0.5 mg/L for 30 min can effectively kill the virus. However, it is crucial to further evaluate the effects of residual chlorine in the environment. Reports indicate that a chlorine concentration of 0.019 mg/L poses an acute toxicity risk to freshwater organisms (https://www.epa.gov/wqc/national-recommended-water-quality-criteria-aquatic-life-criteria-table, accessed on 1 October 2020). In our study, the effective chlorine level was 0.014 mg/L, making it suitable for chronic exposure. After 7 days of exposure, non-targeted absolute quantitative lipid detection revealed a significant reduction in six lipid categories. Over 700 differentially expressed genes (DEGs) were observed that were primarily associated with lipid metabolism and glucose metabolic pathways.

Energy metabolism can often be disrupted by environmental factors such as increased levels of pollutants [34,35]. In this study, we measured several physical indices and energy metabolism-related genes (Table 1 and Figure 1). We found that NaClO exposure significantly reduced glucose (GLU) levels at higher concentrations. Additionally, we examined the genes encoding key enzymes in the glycolysis pathway and observed that glucokinase (*GK*) was overexpressed following NaClO exposure (Figure 1B). Interestingly, pyruvic acid levels decreased after exposure. We hypothesize that, to meet energy demands, more pyruvate was directed into the tricarboxylic acid (TCA) cycle to sustain the organism. However, pyruvate kinase (*PK*) expression was not upregulated, which likely explains the significant reduction in pyruvic acid. It is speculated that zebrafish larvae derive energy from yolk consumption and maintain their energy balance through glucose and pyruvic acid metabolism. Similarly, numerous studies have reported that exposure to hazardous substances increases the expression of lipid-related genes [36,37]. The two genes, *FAS* and *ACCL*, play an important role in the synthesis of fatty acids [37,38]. We found that the expression of *ACC1* and *FAS* significantly changed after exposure to high concentrations of NaClO, (Figure 1A). Additionally, we observed a significant increase in the expression of *PPAR-γ* (Figure 1A). PPAR-γ is a key gene that regulates lipid metabolism and adipogenesis in the liver [39,40]. Research has also shown that the modulation of the nuclear receptor *PPAR-γ* is involved in most cases of obesity in mammals [41]. Therefore, we hypothesized that the increased expression of *PPAR-γ* in our study may induce lipid metabolism disorder. During early embryonic development in zebrafish, larval growth depends on the lipids stored in the yolk, and impairments to the yolk sac may affect nutrient uptake during this critical developmental stage [42]. Cholesterol, as a critical component of cells, plays a vital role in maintaining cellular function. Abnormal cholesterol levels can adversely affect zebrafish growth. The genes *cyp51* and *HMGCR* play an important role in cholesterol synthesis by demethylation and rate-limiting, respectively [43]. Cholesterol 7α-hydroxylase (*cyp7a1*), another rate-limiting enzyme, regulates cholesterol synthesis through feed-forward regulation by oxysterols and feedback regulation by bile acids. In the present study, the expression of the genes *cyp51*, *HMGCR*, and *cyp7a1* was found to be up-regulated (Figure 1C). Collectively, the results of RT-qPCR and the lipid composition analysis suggest that the increased expression of these genes may result from negative feedback due to the decrease in lipid composition in NaClO-treated zebrafish.

Unlike conventional methods, LC-MS-based lipidomics facilitates the concurrent identification and quantification of over 1000 lipid molecules, thereby directly elucidating the interconnections between phenotypes and the underlying mechanisms [44]. An untargeted lipidome analysis was conducted to investigate the alterations in lipid profiles following exposure to NaClO. A total of 174 lipid molecules were detected, encompassing 6 major groups of lipids, including FA, GL, GP, SP, ST, and SL (Figure 2). These lipids play crucial roles in metabolism and physiological regulation during organismal growth [45]. To evaluate the differentially expressed lipids and to illustrate the relationship between samples and lipid expression patterns more comprehensively, we performed hierarchical clustering based on the differential expression levels of the 174 lipids, which included 137 down-regulated and 37 up-regulated metabolites (Figure 3C). Lipid analysis revealed elevated concentrations of glycerophospholipids, particularly PE (Figure 3E,F). Glycerophospholipids, a basic component for the development of cell membranes, are also closely linked to energy metabolism [2]. As shown in Figure 3E, PE constituted the major lipid component in both the control and treatment groups. We hypothesized that the content of PE decreased due to energy metabolism disorder. Several studies on glycerophospholipids have been performed in the field of environmental toxicology [45]. In a previous study, 18 specific lipid metabolites were used to differentiate cadmium (Cd) and benzopyrene (BP) co-exposed samples from the controls [46]. Zhang et al. identified PCs as latent biomarkers to diagnose cancer [47]. In our study, the content of PC increased significantly following treatment with a high concentration of NaClO, indicating a disruption in glycerophospholipid metabolism in the zebrafish. Furthermore, TGs were identified as the most predominantly altered lipid class at the molecular level, followed by PEs and DGs. Petersen et al. identified TGs as biomarkers for disease diagnosis [48]. TG metabolic disorder can also lead to alterations in hepatic lipids [49]. After treatment with R-(−)-IBU, the concentration of glycerol was significantly down-regulated [45], and our results are consistent with this finding. Overall, these results further indicate that NaClO exposure causes hepatic lipid metabolism disorder.

Transcriptomic analysis is widely used in toxicology to reflect changes in biomolecular indicators, providing insights into the mechanisms of toxicity [50,51,52]. In this study, the transcriptome data revealed that significant changes were induced by NaClO exposure. A total of 721 genes were differentially expressed, with 437 being up-regulated and 284 down-regulated. KEGG enrichment analysis indicated that NaClO exposure impacted energy metabolism, including lipid, amino acid, and glucose metabolism. TG metabolism relies not only on exchanges with systemic circulation but also on endogenous lipolysis, serving as a key regulator of systemic homeostasis [53]. Our results demonstrated an overall reduction in lipid storage in zebrafish larvae (Figure 2 and Figure S4). In the transcriptome analysis, several pathways were found to be related to the lipid metabolic pathways such as the PPAR signaling pathway and fatty acid metabolism, which is associated with TG mobilization [34]. These findings are consistent with another study indicating that PCB exposure in the F0 generation enhances TG mobilization, due to increased energy demands [44]. The genes related to lipid metabolism in the transcriptome were individually subjected to enrichment analysis, resulting in the annotation of a total of 25 genes (Figure 5A). Several key genes were selected to verify the transcriptome results, and the qRT-PCR results are consistent with the above analysis. Apoa1b, an apolipoprotein, is involved in lipid and hormone metabolism and is critical for lipid transport [54]. Previous studies have shown that *Apoa1b* can be modulated in response to environmental induction [55]. *Msmo1* is involved in cholesterol biosynthesis [56]. Wong et al. showed that exposure to fluoxetine downregulated the expression of this gene, which is consistent with our finding [57]. Fabp is an intracellular lipid-binding protein; this group includes fabp1b.1 and fabp1b.2 and is highly expressed in the tissues involved in lipid metabolism [58]. The level of fabp1b.1 expression increased significantly after exposure to a high concentration of NaClO. ACADL catalyzes chain-specific fatty acid dehydrogenation [35,59]; its over-expression indicates an increase in fatty acid β-oxidation. Based on integrated transcriptomics, we concluded that NaClO exerted general toxicity on exposed zebrafish embryos. In summary, based on our study, we found that high concentrations of NaClO had a significant impact on aquatic life. Therefore, optimization of the dosage of disinfectants should be considered during the water treatment process to avoid the environmental and health risks caused by their excessive use. For example, when treating wastewater containing NaClO, monitoring of the residual concentration of disinfectants in the discharge water should be strengthened to ensure that contents are below the threshold. Secondly, alternative disinfectants such as ultraviolet treatment or ozone disinfection could also be considered, to reduce the use of high-dose chemical disinfectants and, thus, reduce the environmental burden.

## 5. Conclusions

In conclusion, our findings indicate that high doses of NaClO (300 μg/L) induced lipid metabolism disorder in larval zebrafish. First, based on lipidomics, a total of 44 characteristic lipids were identified; NaClO exposure decreased the content of 6 lipids, including fatty acyls, glyceride, glycerophospholipids, sphingolipids, sterol lipids, and saccharolipids, especially TG and PE. Based on lipidomics, transcriptomic analysis was performed to assess its relationship with the metabolic pathways. After exposure to NaClO, lipid metabolism-related pathways, such as the PPAR signaling pathway, and fatty acid metabolism were enriched. Furthermore, the increase in the transcriptional levels of *ACC1*, *FAS*, and *PPAR-γ* mainly compensated for the imbalance caused by lipid reduction at the gene level to achieve homeostasis in vivo. Thus, these results indicate that NaClO induced lipid metabolism disorder in zebrafish larvae. Lipid metabolism plays an important role in the growth of larval zebrafish, and other effects of disinfectants need to be explored further. Our findings emphasize the ecological risks of the extensive use of chlorine-containing disinfectants for disease control.

## Figures and Tables

**Table 1:** Effects of NaClO exposure on the biochemical indicators of zebrafish larvae.

Biochemical Indicators	NaClO (μg/L)			
	Con	30	100	300
TC (mmol/gprot)	0.0811 ± 0.0021	0.0779 ± 0.0011	0.0824 ± 0.0022	0.0773 ± 0.0018
TG (mmol/gprot)	0.0125 ± 0.0014	0.0127 ± 0.0006	0.0094 ± 0.0007	0.0101 ± 0.0014
Glucose (mmol/gprot)	0.1439 ± 0.0052	0.1524 ± 0.0119	0.1207 ± 0.0213 *	0.1264 ± 0.0187 *
Pyruvate (μmol/gprot)	0.0034 ± 0.0011	0.0043 ± 0.0020	0.0023 ± 0.0002	0.0013 ± 0.0001 **
LDL (mmol/gprot)	0.0478 ± 0.0028	0.0546 ± 0.0017	0.0514 ± 0.0014	0.0489 ± 0.0008

The presented values are the mean ± SEM (n = 6), * *p* < 0.05; ** *p* < 0.01.

## Data Availability

Data will be made available on request.

## Supplementary Materials

The following supporting information can be downloaded at: https://www.mdpi.com/article/10.3390/toxics12100718/s1, Text S1: Lipidomic analysis; Table S1: The primer sequence pairs used in the qRT-PCR; Table S2: Significantly changed lipids in top 10 lass (*p* value < 0.05, log2FoldChange|>2); Figure S1: Lipid correlation analysis in control group; Figure S2: Lipid correlation analysis in 300 μg/L NaClO-treated group; Figure S3: Potential biomarkers detected in larval zebrafish. A. Significant differences in the proportion of lipid molecules; B. Top 10 significantly differentially expressed lipid molecules; C. Some potential biomarkers detected in larval zebrafish (*p* < 0.05, foldchange ≥ 4); Figure S4: GO-MF and GO-CC analysis of DEGs in larval zebrafish after exposure to 100 μg/L 6PPD for 7 days.
