# Supplementary material for: Sodium Hypochlorite (NaClO) Disturbed Lipid Metabolism in Larval Zebrafish (Danio rerio), as Revealed by Lipidomics and Transcriptomics Analyses
Source: Toxics. 2024 Sep 30;12(10):718. doi: 10.3390/toxics12100718 (PMC11510785; doi:10.3390/toxics12100718)

Lipidomics perturbations in larval zebrafish (*Danio rerio*) after exposure to sodium hypochlorite (NaClO)

Text S1：Lipidomic analysis
Table S1 The primer sequence pairs used in the qRT-PCR.
Table S2 Significantly changed lipids in top 10 lass(p.value<0.05, log2FoldChange｜>2)
Figure S1 Lipid correlation analysis in control group
Figure S2 Lipid correlation analysis in 300 μg/L NaClO-treated group
Figure S3 Potential biomarkers detected in larval zebrafish.
Figure S4 GO-MF and GO-CC analysis of DEGs in larval zebrafish after exposure to 100 μg/L 6PPD for 7 days.

Text S1

Quality control (QC) samples

A QC sample was prepared by mixing the aliquots of all the individually treated samples and used to evaluate instrument stability and data reliability. A QC sample was injected before injecting samples to assure analytical repeatability and placed randomly in batches to eliminate the influences of injection order.

Sample pretreatment

The samples were extracted using a series of organic solvents. Firstly, taken an appropriate sample, added 200 μL water and 20 μL internal lipid standard mixture, and then, swirled and mixed. And then, added 800 μL of MTBE，240 μL of pre-cooled methanol, and sonicated for 20 minutes. After stewing at room temperature for 30 minutes, the sample was centrifuged on the centrifuge at 14000 g for 15 min at 4 °C. Taken the upper organic phase and evaporated to dryness under nitrogen at room temperature. Finally, the residue was dissolved in 200 μL 90% isopropyl alcohol and acetonitrile.

Chromatography spectrometry

The extracts of the zebrafish samples were analyzed using Ultra performance liquid chromatography system (UHPLC Nexera LC-30A). The system with a C18 column (2.1 mm × 100 mm, 1.7 μm) was used and maintained at 45 °C. The mobile phase consisted of acetonitrile and water (6:4 v/v) (solvent A), and acetonitrile and isopropanol (1:9 v/v) (solvent B). The gradient elution program was as follows: 0–2.00 min, 30% B; 2.10 min, 50% B; 2-25 min, 30%-100% B; 25-35 min, 30% B. The temperature of the injection chamber maintained at 10 °C.

Mass spectrometry

Using ESI+ and ESI− ion modes for detection. The EIS source conditions are as follows: Heater Temp 300°C，Sheath Gas Flow rate 45arb, Aux Gas Flow Rate15 arb, Sweep Gas Flow Rate 1arb, spray voltage 3.0KV, Capillary Temp 350°C, S-Lens RF Level 50%, MS^1 scan ranges:200-1800.

Data analysis

LipidSearch was used for peak identification, peak extraction and lipid identification of lipid molecules and internal standard lipid molecules. The main parameters are as following:precursor tolerance:5ppm, product tolerance:5ppm,product ion threshold:5%.

Table S1. The primer sequence pairs used in the qRT-PCR.

Gene	**Sequence of the forward primers (5’-3’)**	**Sequence of the reverse primers (5’-3’)**
PPAR-α	CTGCGGGACATCTCTCAGTC	ACCGTAAACACCTGACGACG
PPAR-γ	CCTGTCCGGGAAGACCAGCG	GTGCTCGTGGAGCGGCATGT
pepckc	ATCACGCATCGCTAAAGAGG	CCGCTGCGAAATACTTCTTC
ACO	ACCTTTGGGATCATGGACTG	TGTTGTTTCGTGCCTCAAAG
APOA	AGCTGCACAATCCTCGTCTGC	TGCAGGAGCCTGTGTTTGCCT
UCP2	GAACTGTGCTGTGGCATGTG	TCGTTTCAGCTGCTCGTAA
ACC1	GCGTGGCCGAACAATGGCAG	GCAGGTCCAGCTTCCCTGCG
FAS	GGAGCAGGCTGCCTCTGTGC	TTGCGGCCTGTCCCACTCCT
PK	TCCTGGAGCATCTGTGTCTG	GTCTGGCGATGTTCATTCCT
GK	GCTGTGAAGTCGGCATGATA	CTTCAACCAGCTCCACCTTAC
HK1	ACTTTGGGTGCAATCCTGAC	AGACGACGCACTGTTTTGTG
cyp51	GCTCGGAGACACTCAGACACATCTT	AGCAGAACTGAAGTCAGGCTCATCT
cyp7a1	CCTTCCTTGGCTGTGCTCTTCAGTT	AGGTGCCTTCCTTGACGGATGACA
hmccra	TCGTGGAGTGCCTGGTGATTGGT	TGGGTCTGCCTTCTCTGCTCTCTC
LDLR	GCCAGCAAGGCCTGCAAAGC	CTTCAGGCGGGGGATGACGC
cyc3c3	GGGAATAAGTGTGTTTTCAAGGTCAATTATGA	GGTTGTTCACTTGTGGTGTCTTTCGC
apoa1b	CTCGCCCTCACCGTATTCCT	CGAACTTCTGGAGGGCCTTG
msmo1	TCAGCATCCCTTATGACTGG	AATGGAGAAGTGAAGTCGTGA
fabp1b.1	AAGCTGAAGGTGGTGCTGAACA	CACGTTTGCTGATGCGCTTGTA
acadl	TTTACTGGGAGTTTACACACC	CTTTAGAGCCATAGTGACTGATA

Table S2. Significantly changed lipids in top 10 lass(p.value<0.05, log2FoldChange丨>2)

Class	LipidIon	Faty Acid	Ion Formula	RT.Time	log2FC	Pvalue	regulated
	TG(16:1e_20:5_22:6)+Na	(16:1e_20:5_22:6)	C61 H96 O5 Na1	16.173	-2.1083	0.02333	down
	TG(16:1_20:5_22:6)+NH4	(16:1_20:5_22:6)	C61 H98 O6 N1	15	-2.1276	0.04041	down
	TG(16:1_20:5_20:5)+NH4	(16:1_20:5_20:5)	C59 H96 O6 N1	14.898	-2.1449	0.02888	down
	TG(20:4e_18:0_20:4)+NH4	(20:4e_18:0_20:4)	C61 H108 O5 N1	16.881	-2.1791	0.00147	down
	TG(16:0e_18:1_18:2)+NH4	(16:0e_18:1_18:2)	C55 H106 O5 N1	17.257	-2.2244	0.03518	down
TG	TG(20:3_13:0_20:3)+Na	(20:3_13:0_20:3)	C56 H96 O6 Na1	15.526	-2.2271	0.03827	down
	TG(14:0_10:3_20:4)+NH4	(14:0_10:3_20:4)	C47 H80 O6 N1	2.9322	-2.2601	0.02856	down
	TG(20:5_20:5_20:5)+NH4	(20:5_20:5_20:5)	C63 H96 O6 N1	14.151	-2.3157	0.02911	down
	TG(20:5_17:1_20:5)+NH4	(20:5_17:1_20:5)	C60 H98 O6 N1	15.239	-2.4109	0.01152	down
	TG(16:0_10:1_12:4)+H	(16:0_10:1_12:4)	C41 H69 O6	11.428	-2.7005	0.04686	down
	TG(16:1e_18:0_18:1)+NH4	(16:1e_18:0_18:1)	C55 H108 O5 N1	17.411	-3.0392	0.02399	down
	PE(33:1_18:0)+H	(33:1_18:0)	C56 H111 O8 N1 P1	16.333	-2.0153	0.0492	down
	PE(18:0_22:6)+H	(18:0_22:6)	C45 H79 O8 N1 P1	17.494	-2.0673	0.01815	down
	PE(35:2)+H	(35:2)	C40 H77 O8 N1 P1	9.332	-2.4884	0.02051	down
	PE(31:0)+H	(31:0)	C36 H73 O8 N1 P1	8.433	-2.6274	0.0303	down
	PE(40:7e)+Na	(40:7e)	C45 H78 O7 N1 P1	10.275	-2.7671	0.04359	down
	PE(26:6e)-H	(26:6e)	C31 H51 O7 N1 P1	6.3678	-2.9566	0.03479	down
	PE(39:3)+Na	(39:3)	C44 H82 O8 N1 P1	7.383	-2.9824	0.04667	down
PE	PE(9:0_9:0)-H	(9:0_9:0)	C23 H45 O8 N1 P1	2.009	-2.9988	0.02427	down
	PE(51:2)+H	(51:2)	C56 H109 O8 N1 P1	16.189	-3.0291	0.0217	down
	PE(33:0_16:1)+H	(33:0_16:1)	C54 H107 O8 N1 P1	16.355	-3.0629	0.01166	down
	PE(26:5)-H	(26:5)	C31 H51 O8 N1 P1	5.5762	-3.1575	0.02261	down
	PE(53:2)+H	(53:2)	C58 H113 O8 N1 P1	16.463	-3.284	0.03085	down
	PE(33:0_18:0)+H	(33:0_18:0)	C56 H113 O8 N1 P1	16.495	-3.2978	0.02802	down
	PE(49:2)+H	(49:2)	C54 H105 O8 N1 P1	16.189	-3.3616	0.02028	down
DG	DG(40:2)+Na	(40:2)	C43 H80 O5 Na1	14.504	-2.246	0.01607	down
	CL(18:3_20:4_18:2_18:1)-	(18:3_20:4_18:2_18:1)	C83 H141 O17 P2	15.41	-2.2077	0.04385	down
	CL(76:1)-2H	(76:1)	C85 H162 O17 P2	10.76	-2.3941	0.03638	down
	CL(78:3)-2H	(78:3)	C87 H162 O17 P2	10.227	-2.7023	0.02458	down
CL	CL(70:3)-2H	(70:3)	C79 H146 O17 P2	9.796	-3.1463	0.00621	down
	CL(82:9)-2H	(82:9)	C91 H158 O17 P2	9.057	-3.3035	0.03686	down
	CL(68:4)-2H	(68:4)	C77 H140 O17 P2	8.351	-3.4159	0.04646	down
	CL(80:6)-2H	(80:6)	C89 H160 O17 P2	4.7886	-4.0495	0.04884	down
PC	PC(21:2)+H	(21:2)	C29 H55 O8 N1 P1	2.558	-6.8581	0.02293	down
Cer	Cer(m38:1+O)+HCOO	(m38:1+O)	C39 H76 O5 N1	13.459	3.18363	0.00701	up
PS	PS(38:3e)-H	(38:3e)	C44 H81 O9 N1 P1	7.233	2.1656	0.01082	up
SPH	SPH(m20:0)+H	(m20:0)	C20 H44 O1 N1	2.909	-2.0315	0.0162	down
	WE(5:0_16:1)+NH4	(5:0_16:1)	H44 C21 O2 N1	3.3529	-2.2711	0.04046	down
WE	WE(2:0_20:3)+NH4	(2:0_20:3)	H42 C22 O2 N1	2.3292	-2.5459	0.00379	down
	WE(2:0_20:2)+NH4	(2:0_20:2)	H44 C22 O2 N1	2.7251	-2.7522	0.0346	down
PI	PI(43:2)+Na	(43:2)	C52 H97 O13 N0 P1	14.17	-2.2976	0.00402	down

Figure S1. Lipid correlation analysis in control group

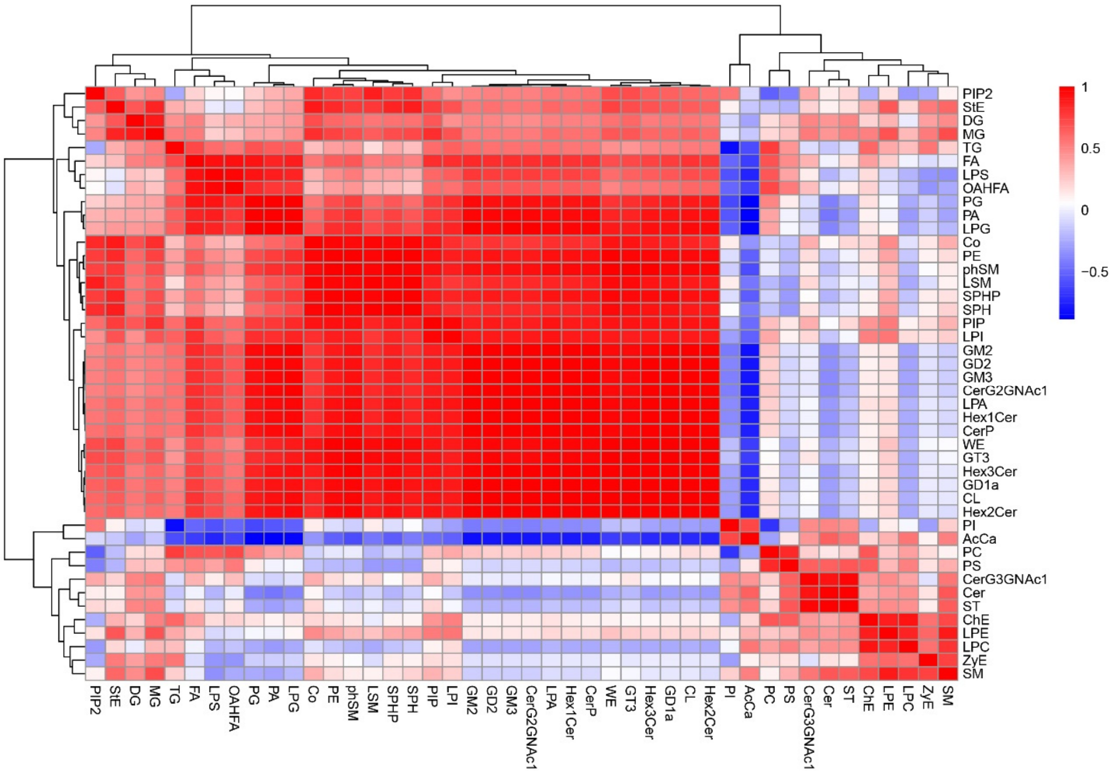

Figure S2. Lipid correlation analysis in 300 μg/L NaClO-treated group

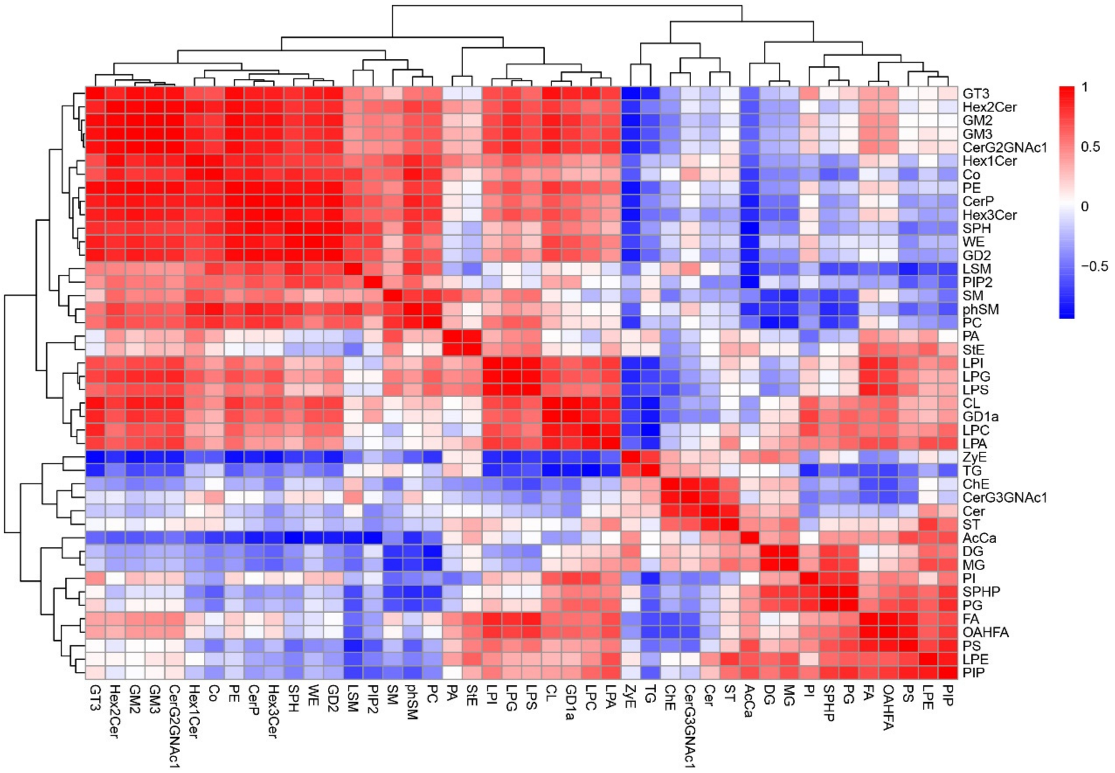

Figure S3. Potential biomarkers detected in larval zebrafish. A. Significant differences in the

proportion of lipid molecules; B. Top 10 significantly differentially expressed lipid molecules;

C. Some potential biomarkers detected in larval zebrafish (p < 0.05, foldchange ≥ 4).

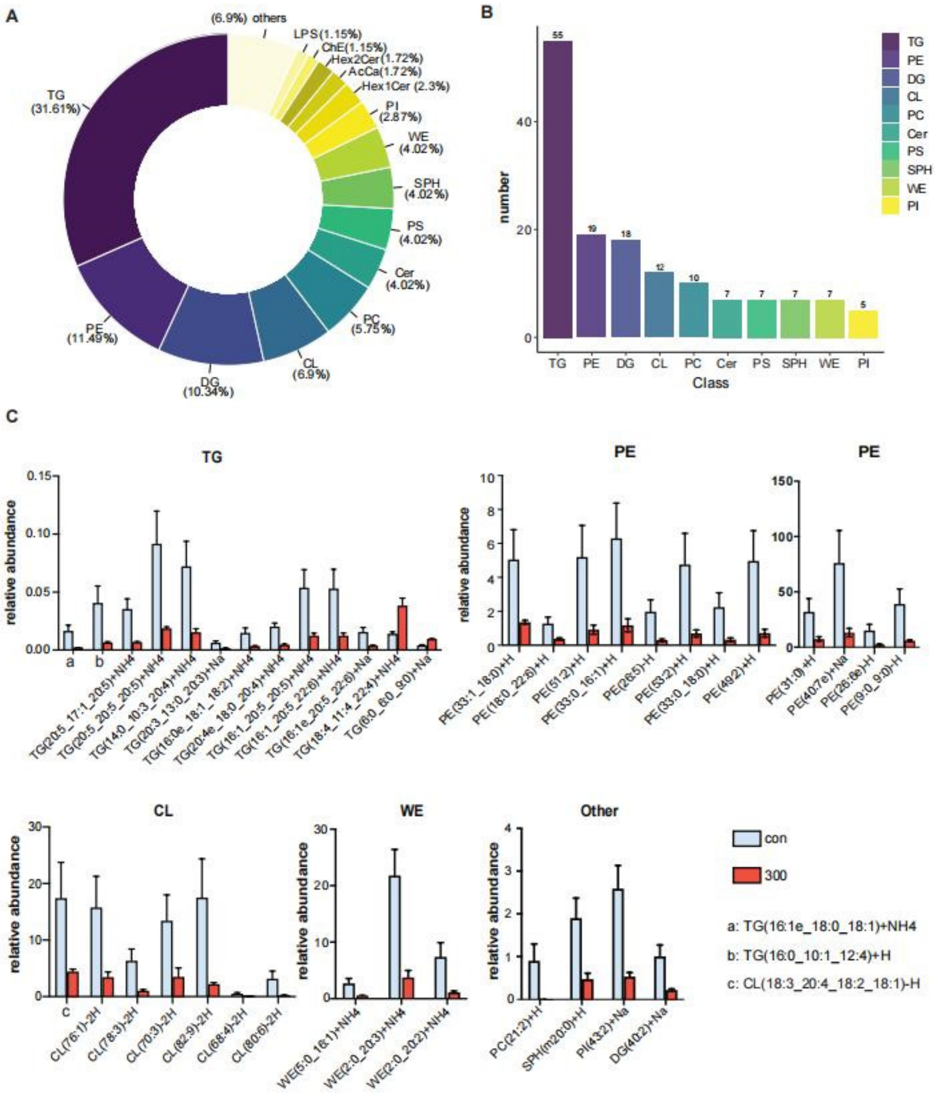

Figure S4. GO-MF and GO-CC analysis of DEGs in larval zebrafish after exposure to 300 μg/L NaClO for 7 days.

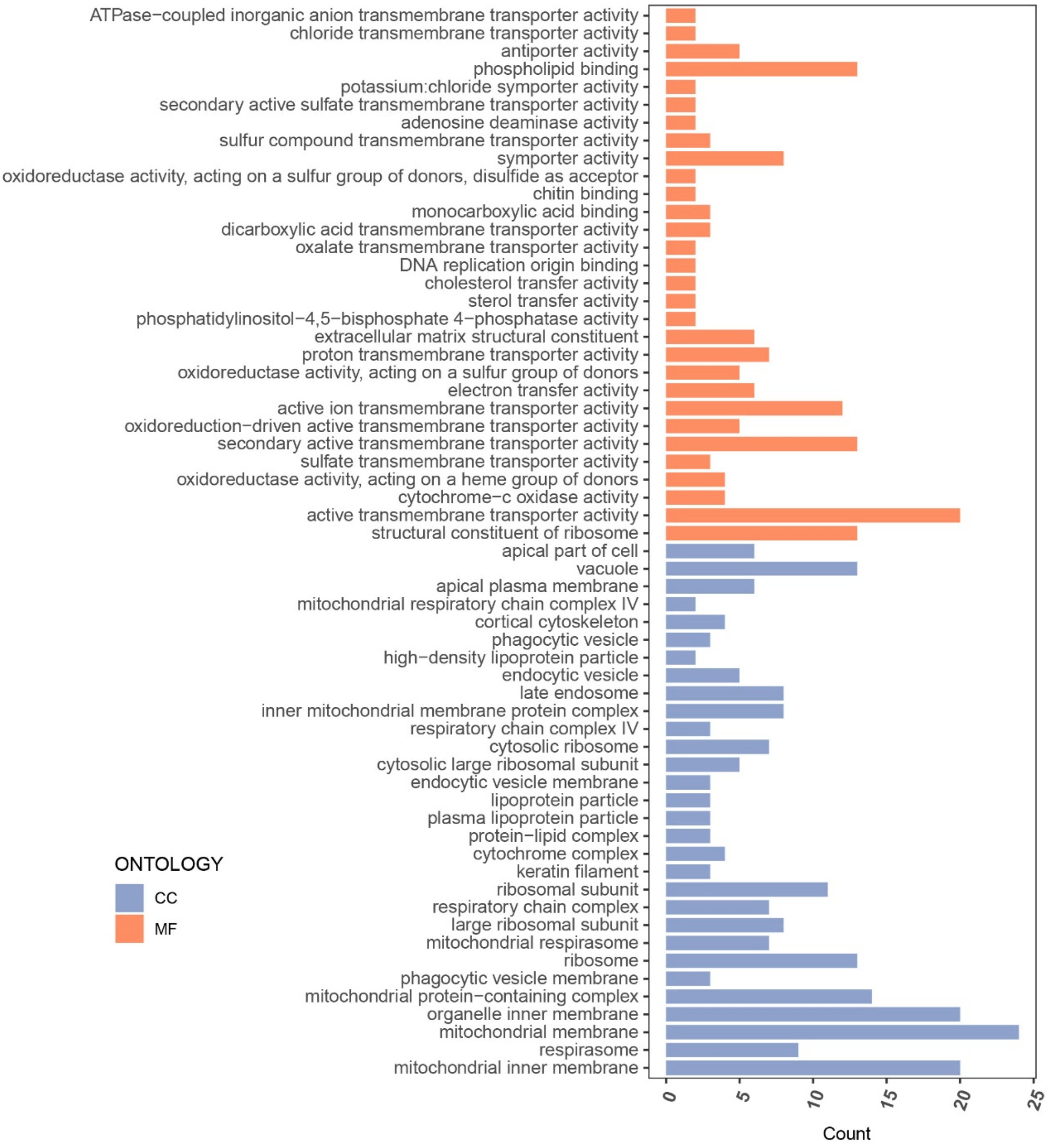

Supplement: Supplementary file 1 [file toxics-12-00718-s001.zip › toxics-3192037-supplementary.pdf]
